# Selection of single-nucleotide polymorphisms in disease association data

**DOI:** 10.1186/1471-2156-6-S1-S93

**Published:** 2005-12-30

**Authors:** Jungnam Joo, Xin Tian, Gang Zheng, Jing-Ping Lin, Nancy L Geller

**Affiliations:** 1 Office of Biostatistics Research, National Heart, Lung and Blood Institute, Bethesda, 6701 Rockledge Dr. MSC 7938, Maryland 20892, USA

## Abstract

We studied several methods for selecting single-nucleotide polymorphisms (SNPs) in a disease association study. Two major categories for analytical strategy are the univariate and the set selection approaches. The univariate approach evaluates each SNP marker one at a time, while the set selection approach tests disease association of a set of SNP markers simultaneously. We examined various test statistics that can be utilized in testing disease association and also reviewed several multiple testing procedures that can properly control the family-wise error rates when the univariate approach is applied to multiple markers. The set association methods were then briefly reviewed. Finally, we applied these methods to the data from Collaborative Study on the Genetics of Alcoholism (COGA).

## Background

Due to the abundance and utility of single-nucleotide polymorphism (SNP) markers in the fine-mapping of complex traits, a growing amount of current genetic research focuses on the analyses of SNP data. Such analyses typically involve association, in which differences in allele or genotype frequencies of SNPs near or within candidate genes between affected and unaffected individuals are tested. To localize disease susceptibility genes (loci), thousands of SNPs are usually investigated and the main question is how to identify disease-associated SNP markers among a large pool.

A simple approach that is commonly used is to evaluate one SNP at a time. In this analytical strategy, each SNP is tested with appropriate testing procedures, such as Pearson's chi-square test and Cochran-Armitage (CA) trend test, and those SNP markers with a significant disease association are identified. Current technology, however, can genotype on the order of 100,000 SNPs at a time. Even with a preliminary genome scan, such as linkage analysis, which can restrict the chromosomal region to reduce the number of SNPs for investigation, often a large number of SNPs are tested simultaneously. Therefore, investigators are at great risk of false-positive findings. Various methods for marker selection with consideration of multiple comparisons are available. Dudoit et al. [1,2] summarized a number of procedures that control different type I error rates, such as family-wise error (FWER) and false discovery rates (FDR) [3].

For a complex trait, however, several markers, each with a rather small effect, might act together to contribute to disease susceptibility. In this case, marker-by-marker approaches often fail to find significance. Recently, several investigators incorporated the multigenic nature of complex traits in selecting SNPs for association [4,5]. One promising approach has been proposed by Hoh et al. [6], which performs a simultaneous significance test on a set of possibly interacting SNP markers while controlling the genome-wide significance level via permutation procedures.

In this study, we describe different strategies for selecting SNPs in a disease association study and apply them to the Collaborative Study on the Genetics of Alcoholism (COGA) data.

## Methods

### Measures for disease association

#### Allelic association and Hardy-Weinberg disequilibrium

To measure the extent of the association for a given SNP, Hoh et al. [6] proposed a statistic that combines several sources of information, such as allelic association (AA) and Hardy-Weinberg disequilibrium (HWD). In a 2 × 2 table with rows corresponding to cases and controls, and columns corresponding to SNP alleles, the *χ*^2 ^statistic can be utilized as a measure for AA. HWD can also be computed using *χ*^2 ^for deviation from Hardy-Weinberg equilibrium based on the affected individuals only. Let *a*_*i *_and *u*_*i *_be the AA statistic and HWD for association of the *i*^th ^SNP, respectively. The product of these two statistics, *a*_*i *_× *u*_*i*_, is used to measure the effects of AA and HWD for association. We denote this test statistic as AA × HWD. Hoh et al. [6] used trimming for markers with extremely high values of HWD. They first find the number *d *of largest HWD values (for example, using 99^th ^percentile of the *χ*^2 ^distribution) based on control individuals, and *d *HWD values are set to zero in the further analysis.

#### Robust linear trend tests-MERT and MAX

Two robust tests, the maximin efficiency robust test (MERT) and the maximal test (MAX) are useful in detecting disease-associated markers when the underlying genetic model is unknown. Suppose we have a family of optimal test statistics {*Z*_*i *_: *i *∈ Λ}, where Λ = {1, 2, ..., *k*}is an index of *k *underlying models. For example, using the CA trend test, *Z*_*x*_, *x *= 0, 1/2, 1, are optimal test statistics for the recessive, additive, and dominant models, respectively [7]. Assume that under the null hypothesis, each *Z*_*i *_asymptotically follows a standard normal distribution and that their correlation matrix under the null hypothesis of no disease association is given by . Closed forms of the test statistics and correlations for the CA-trend test in case-control studies can be found in Friedlin et al. [8]. From Gastwirth [9], MERT can be written as a linear combination of two tests with the minimum correlation. Suppose that the minimum correlation  is reached at the two tests  and , *i*_1_, *i*_2 _∈ Λ. Then, a linear combination of the extreme pair given by



which asymptotically follows a standard normal distribution under the null hypothesis.

When the minimum correlation ρ_0 _is small, MERT may not be powerful. Freidlin et al. [10] suggested the use of a maximal statistic (MAX) when ρ_0 _< 0.50 and showed that the MAX and MERT have similar power when ρ_0 _≥ 0.75. Several versions of MAX tests are possible but here we focus on *Z*_MAX _= max(, *Z*_MERT_, ) for a one-sided test and *Z*_MAX _= max(||, |*Z*_MERT_|, ||) for a two-sided test.

### Multiple testing

Dudoit et al. [1] provided multiple testing procedures which strongly control the FWER for gene expression data and which are directly applicable to disease association data with multiple markers. The Bonferroni single-step adjusted *p*-value is a well known procedure for dealing with multiple testing. While it is easy to calculate, this method is extremely conservative. The improvement in power can be achieved by step-wise procedures such as Holm's procedure. To take into account the dependence structure between test statistics, Westfall and Young's [11] step-down min*P *or step-down max*T *adjusted *p*-values are useful. Since the joint distribution of the test statistics is usually unknown, resampling methods can be used to estimate these adjusted *p*-values.

### Set association approach

Hoh et al. [6] provided a method that tests the disease-association of a set of markers instead of testing each SNP separately. In their method, the sum of test statistics over a suitable set of markers is first formed to combine the evidence for association. Permutation procedures are then used to evaluate *p*-values associated with each sum and the overall type I error. The following summarizes the set association approach of Hoh et al. [6].

1) Order test statistics t_*i*_, *i *= 1, ..., *m*, so that |*t*_(1)_| ≥ |*t*_(2)_| ≥ ... ≥ |*t*_(*m*)_|.

2) For a fixed *N *≤ *m*, take sums with an increasing number of terms, starting with the most significant markers, such that *S*(*n *= 1) = |*t*_(1)_|, *S*(*n *= 2) = |*t*_(1)_| + |*t*_(2)_|, ..., *S*(*n *= *N*) = |*t*_(1)_| + ... + |*t*_(*N*)_|.

3) Generate the permutation samples from the original sample (permuting labels of cases and controls) under the null hypothesis of no association and evaluate the *p*-value of each sum. Take the minimum *p*-value (min*P*).

4) Generate other permutation samples from the original sample under the null hypothesis of no association. To obtain the *p*-value corresponding to each permutation sample, repeat the above 3 steps by regarding each permutation sample as the original.

5) Evaluate the overall significance level of (min*P*).

### Study subjects and genetic markers

The COGA data provide alcoholism diagnosis on 1,614 individuals from 143 families. We focus on two categories for the alcoholism diagnosis (aldx1), "affected" as a case and "purely unaffected" as a control, and we used all 609 cases and 261 controls whose SNP data were available. From the preliminary genome scan by linkage analysis (Lin and Wu [12]), one candidate gene cluster, alcohol dehydrogenase, on chromosome 4 was identified. Alcohol dehydrogenase catalyzes the rate-determining reaction in ethanol metabolism. Genetic studies of diverse ethnic groups have firmly demonstrated significant allelic associations between alcohol dehydrogenase genes and alcoholism. Therefore, we restrict our analysis to SNPs located near this gene cluster. Because the SNPs are evenly distributed in the entire genome but not densely genotyped near any genes, we found two SNPs (rs749407, rs980972) within the cluster and we selected two additional SNPs (rs1037475, rs1491233) flanking each side from the Illumina SNP data.

## Results

Table 1 presents the results from the univariate method for testing association using four test statistics, *χ*^2^, AA × HWD, MERT, and MAX. The unadjusted *p*-values for AA × HWD were obtained via permutation with 20,000 replicates and the *p*-values for MAX were calculated based on 20,000 simulations. In Hoh et al. [6], unusually large HWD values were trimmed based on HWD in control individuals. Because we did not find any SNP markers whose HWD value was larger than their suggested cut-off value (the 99^th ^percentile for a *χ*^2 ^distribution with 1 degree of freedom) we did not need trimming in our analysis. The disease-association of rs1037475 is significant based on most of the test statistics with correction for multiple testing. The smallest correlations between linear trend tests for recessive and dominant models for all four SNP markers were less than 0.4, and therefore MAX may be more efficient than MERT [10]. As expected, Westfall and Young's step-down method is less conservative than Holm's method, which in turn is less conservative than the Bonferroni correction. One exception is found when we used AA × HWD. We found that even though rs1037475 has the maximum observed test statistic (19.685), other markers have a larger chance of having a test statistic greater than 19.685 in the permutation samples. We do not know why this happened, but it shows that the test statistic AA × HWD is rather unstable in the permutation procedure. The SNP marker rs1037475 shows a significant disease association using the *χ*^2 ^and MAX tests. The other three markers failed to show a significant association.

**Figure 1:**
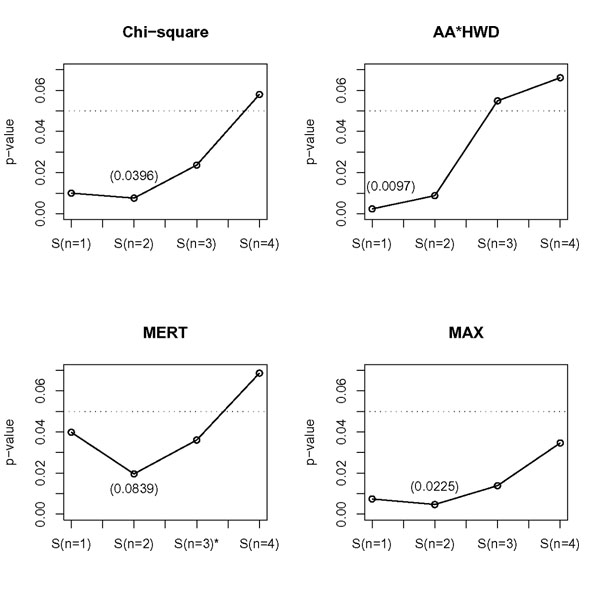
**Significance level of the set association approach using different test statistics**. *S*(*n *= 1): SNP marker rs1037475 *S*(*n *= 2): SNP markers rs1037475, rs980972 *S*(*n *= 3): SNP markers rs1037475, rs980972, rs1491233 *S*(*n *= 3)*: SNP markers rs1037475, rs980972, rs749407 *S*(*n *= 4): all four markers

Figure 1 summarizes the result from the set association approach. Because there were only four markers under investigation, we considered the sum of test statistics up to all four SNP markers. We performed 20,000 permutations to obtain corresponding *p*-values for each of 10,000 permutation samples. The order of SNP markers included in the sum statistics based on the univariate test statistics is rs1037475, rs980972, rs1491233, rs749407, except for MERT, where rs1491233 and rs749407 are switched. Using *χ*^2^, MERT, and MAX, the smallest *p*-value is reached at *S*(*n *= 2), which is the sum statistic of rs1037475 and rs980972. For AA × HWD, the smallest *p*-value is obtained at *S*(*n *= 1). The overall significance levels of these smallest *p*-values (adjusted for multiplicity) are 0.0396, 0.0097, 0.0839, and 0.0225 for *χ*^2^, AA × HWD, MERT, and MAX, respectively. Only MERT failed to reach the global significance level. Using univariate analyses, rs980972 has rather negligible effect. However, the effect of rs980972 combined with rs1037475 became significant using the set association approach.

We carried out an additional analysis on a total of 8 SNPs in the nearest area including the above four SNPs. Using the univariate method with Bonferroni and Holm's methods, only AA × HWD found rs1037475 to be significant. None of the methods found significant markers based on Westfall and Young's method. In the set association approach, the smallest *p*-values were reached at *S*(*n *= 1) using *χ*^2 ^and AA × HWD, and at *S*(*n *= 2) using MERT and MAX, where *S*(*n *= 1) corresponds to rs1037475 and *S*(*n *= 2) is the sum of rs1037475 and rs980972. The overall significance levels of these smallest *p*-values were 0.094, 0.022, 0.226, and 0.074, respectively. Again, only AA × HWD reached the overall significance at *α *= 0.05. When we included more SNPs in the analysis (a total of 28), none of the methods found significant markers. By adding SNPs which may not be in linkage disequilibrium with the mutation, the method became extremely conservative.

## Conclusion

In this paper, we studied different strategies to select disease-associated SNP markers when multiple markers are tested. Various test statistics can be utilized to measure the degree of individual association, and using these statistics, the univariate approach combined with an appropriate correction for multiple testing can identify significant markers. However, if several markers are acting together to contribute to the susceptibility of the disease, the set association approach may be useful. In the application to the COGA data, we observed different results using the univariate and set association approaches, that is, a SNP marker with a rather negligible effect using the univariate approach is picked up by the set association approach. An added advantage of the set association methods is their ability to detect interacting loci, though we do not investigate that property here. For a rigorous comparison of the performances between different approaches, further investigation with simulated data would be necessary.

We used only four SNPs in our analysis. In principal, these procedures can also be applied to testing thousand of SNPs as in a genome-wide association study. However, for testing a very large number of SNPs, these procedures can be extremely conservative and computationally intense. As we include more SNPs in the analysis, the methods tend to become very conservative and fail to find any significance. Reducing the number of tests by restricting areas of investigation is one common approach to address the multiple testing problems in genome-wide association studies and the methods described here may be optimal with the reduced data. To take full advantage of the abundant information from a genome-wide SNP map, alternative approaches such as a method for controlling FDR and a sequential type analysis [13] are possible.

The choice of test statistics has a great impact on the testing results. The CA trend test is usually preferable to the *χ*^2 ^test [14,15] and two robust tests, MERT and MAX, provide protection against model misspecification [7,8]. AA × HWD [6] showed quite consistent result using different numbers of SNPs in the analysis. However, its performance was unstable in the permutation procedure. The properties of these test statistics under a variety of genetic models may need further investigation.

The case-control dataset used in this study is a family dataset in which cases and controls could be biologically correlated. The effect of correlated structures between family members in statistical testing leads to an inflated variance due to the positive correlation. Therefore, without considering this factor, inflation in type I error rates may result. In one of our studies using the same dataset [16], we applied the method of Slager and Schaid [17] with modification, in which the correlations of related individuals are incorporated into the CA trend test. While adjusting for the correlations is desirable, we found that the variance inflation is rather minor, and thus in this study, we ignored family structure. The test statistics which incorporate the correlations between family members can also be utilized in the univariate and set association approaches described in this study.

## Abbreviations

AA: Allelic association

CA: Cochran-Armitage

COGA: Collaborative Study on the Genetics of Alcoholism

FDR: False discovery rates

FWER: Family-wise error rate

HWD: Hardy-Weinberg disequilibrium

MAX: Maximal text

MERT: Maximin efficiency robust test

SNP: Single-nucleotide polymorphism

## Authors' contributions

JJ was involved in the design of the study, performed statistical analysis and interpretation of data, and drafted the manuscript. XT, GZ, J-PL and, NLG were involved in the design of the study, statistical analysis, interpretation of data, and revising the manuscript. All authors read and approved the final manuscript.

## Figures and Tables

**Table 1:** Results from the univariate methods

		rs1037475				rs1491233			
			
		*χ*^2^	AA × HWD	*Z*^2^_MERT_	*Z*^2^_MAX_	*χ*^2^	AA × HWD	*Z*^2^_MERT_	*Z*^2^_MAX_
Test statistic		9.299	19.685	4.234	8.842	0.620	1.301	0.380	0.616
*p*-value^1^		0.010	0.002	0.040	0.007	0.734	0.587	0.537	0.674
*p*-value^2^	Bon^3^	0.040	0.008	0.160	0.028	1.000	1.000	1.000	1.000
	Holm	0.040	0.008	0.160	0.028	1.000	0.742	0.888	1.000
	wy^4^	0.037	0.076	0.141	0.025	0.920	0.680	0.542	0.886

		rs749407				rs980972			

Test statistic		0.619	0.929	0.586	0.586	4.900	5.244	3.848	4.820
*p*-value^1^		0.734	0.371	0.444	0.684	0.086	0.136	0.050	0.060
*p*-value^2^	Bon^3^	1.000	1.000	1.000	1.000	0.344	0.544	0.200	0.240
	Holm	1.000	0.742	0.888	1.000	0.258	0.408	0.160	0.180
	wy^4^	0.730	0.526	0.670	0.689	0.234	0.224	0.135	0.156

*p*-value^1^: Unadjusted *p*-value

*p*-value^2^: Adjusted *p*-value

Bon^3^: Bonferroni single-step correction

wy^4^: Westfall and Young's max*T *step-down correction.
